# The high cost of prescription drugs: causes and solutions

**DOI:** 10.1038/s41408-020-0338-x

**Published:** 2020-06-23

**Authors:** S. Vincent Rajkumar

**Affiliations:** The Division of Hematology, Mayo Clinic, Rochester, MN USA

**Keywords:** Public health, Cancer therapy

Global spending on prescription drugs in 2020 is expected to be ~$1.3 trillion; the United States alone will spend ~$350 billion^1^. These high spending rates are expected to increase at a rate of 3–6% annually worldwide. The magnitude of increase is even more alarming for cancer treatments that account for a large proportion of prescription drug costs. In 2018, global spending on cancer treatments was approximately 150 billion, and has increased by >10% in each of the past 5 years^2^.

The high cost of prescription drugs threatens healthcare budgets, and limits funding available for other areas in which public investment is needed. In countries without universal healthcare, the high cost of prescription drugs poses an additional threat: unaffordable out-of-pocket costs for individual patients. Approximately 25% of Americans find it difficult to afford prescription drugs due to high out-of-pocket costs^3^. Drug companies cite high drug prices as being important for sustaining innovation. But the ability to charge high prices for every new drug possibly slows the pace of innovation. It is less risky to develop drugs that represent minor modifications of existing drugs (“me-too” drugs) and show incremental improvement in efficacy or safety, rather than investing in truly innovative drugs where there is a greater chance of failure.

## Causes for the high cost of prescription drugs

### Monopoly

The most important reason for the high cost of prescription drugs is the existence of monopoly^4,5^. For many new drugs, there are no other alternatives. In the case of cancer, even when there are multiple drugs to treat a specific malignancy, there is still no real competition based on price because most cancers are incurable, and each drug must be used in sequence for a given patient. Patients will need *each* effective drug at some point during the course of their disease. There is seldom a question of *whether* a new drug will be needed, but only *when* it will be needed. Even some old drugs can remain as virtual monopolies. For example, in the United States, three companies, NovoNordisk, Sanofi-Aventis, and Eli Lilly control most of the market for insulin, contributing to high prices and lack of competition^6^.

Ideally, monopolies will be temporary because eventually generic competition should emerge as patents expire. Unfortunately, in cancers and chronic life-threatening diseases, this often does not happen. By the time a drug runs out of patent life, it is already considered obsolete (planned obsolescence) and is no longer the standard of care^4^. A “new and improved version” with a fresh patent life and monopoly protection has already taken the stage. In the case of biologic drugs, cumbersome manufacturing and biosimilar approval processes are additional barriers that greatly limit the number of competitors that can enter the market.

Clearly, all *monopolies* need to be regulated in order to protect citizens, and therefore most of the developed world uses some form of regulations to cap the launch prices of new prescription drugs. *Unregulated monopolies* pose major problems. *Unregulated monopoly over an essential product* can lead to unaffordable prices that threaten the life of citizens. This is the case in the United States, where there are no regulations to control prescription drug prices and no enforceable mechanisms for value-based pricing.

### Seriousness of the disease

High prescription drug prices are sustained by the fact that treatments for serious disease are not luxury items, but are needed by vulnerable patients who seek to improve the quality of life or to prolong life. A high price is not a barrier. For serious diseases, patients and their families are willing to pay any price in order to save or prolong life.

### High cost of development

Drug development is a long and expensive endeavor: it takes about 12 years for a drug to move from preclinical testing to final approval. It is estimated that it costs approximately $3 billion to develop a new drug, taking into account the high failure rate, wherein only 10–20% of drugs tested are successful and reach the market^7^. Although the high cost of drug development is a major issue that needs to be addressed, some experts consider these estimates to be vastly inflated^8,9^. Further, the costs of development are inversely proportional to the incremental benefit provided by the new drug, since it takes trials with a larger sample size, and a greater number of trials to secure regulatory approval. More importantly, we cannot ignore the fact that a considerable amount of public funding goes into the science behind most new drugs, and the public therefore does have a legitimate right in making sure that life-saving drugs are priced fairly.

### Lobbying power of pharmaceutical companies

Individual pharmaceutical companies and their trade organization spent approximately $220 billion in lobbying in the United States in 2018^10^. Although nations recognize the major problems posed by high prescription drug prices, little has been accomplished in terms of regulatory or legislative reform because of the lobbying power of the pharmaceutical and healthcare industry.

## Solutions: global policy changes

There are no easy solutions to the problem of high drug prices. The underlying reasons are complex; some are unique to the United States compared with the rest of the world (Table 1).

**Table 1:** Reasons for the high cost of prescription drugs and possible solutions.

Factor contributing to high price	Proposed solutions
*Global policy level*	
Monopoly/oligopoly	Patent reform, including fixed duration of patent protection starting with first approval and prohibiting additional patents on approved drugs that seek to increase patent life Penalties for pay-for-delay schemes and frivolous lawsuits that delay generic or biosimilar entry Expedite approval processes for generics and biosimilars, including reciprocal approval arrangements among countries Nonprofit generic manufacturing
Seriousness of the disease	Greater use of compulsory licensing if negotiations on reasonable price are not successful for life-threatening diseases
Drug development costs	Regulatory reform to minimize the amount of supplemental data needed for approval Harmonize differences in regulatory standards for submission and approval between the United States and Europe Discourage approval of drugs with statistically significant but clinically insignificant benefits
Pharmaceutical lobbying	Transparency in lobbying spending Transparency in funds received by professional and patient organizations from drug manufacturers
*United States policy level*	
Lack of agency with legal authority to regulate prices	Agency that sets value-based ceiling price as currently done in Western Europe must be adopted Medicare authorized to directly negotiate prices Caps on price increases of approved drugs that are under patent protection Permit importation of prescription drugs for personal use
More favorable reimbursement for more expensive drugs	Abolish reimbursement to doctors as a percentage of the price of the drug, and replace with a fixed reimbursement, regardless of drug price.
Costs incurred due to middlemen	Transparency on arrangements between pharmaceutical companies and pharmacy-benefit managers (PBMs) Rebates issued by PBMs are passed on to patients
*Physician level*	
Lack of awareness	Awareness on drug prices, and discuss affordability with patients
Lack of advocacy	Reduce conflicts of interest that prevent physicians and physician organizations from advocating for policies that lower prescription drug costs

### Patent reform

One of the main ways to limit the problem posed by monopoly is to limit the duration of patent protection. Current patent protections are too long, and companies apply for multiple new patents on the same drug in order to prolong monopoly. We need to reform the patent system to prevent overpatenting and patent abuse^11^. Stiff penalties are needed to prevent “pay-for-delay” schemes where generic competitors are paid money to delay market entry^12^. Patent life should be fixed, and not exceed 7–10 years from the date of first entry into the market (one-and-done approach)^13^. These measures will greatly stimulate generic and biosimilar competition.

### Faster approval of generics and biosimilars

The approval process for generics and biosimilars must be simplified. A reciprocal regulatory approval process among Western European countries, the United States, Canada, and possibly other developed countries, can greatly reduce the redundancies^14^. In such a system, prescription drugs approved in one member country can automatically be granted regulatory approval in the others, greatly simplifying the regulatory process. This requires the type of trust, shared standards, and cooperation that we currently have with visa-free travel and trusted traveler programs^6^.

For complex biologic products, such as insulin, it is impossible to make the identical product^15^. The term “biosimilars” is used (instead of “generics”) for products that are almost identical in composition, pharmacologic properties, and clinical effects. Biosimilar approval process is more cumbersome, and unlike generics requires clinical trials prior to approval. Further impediments to the adoption of biosimilars include reluctance on the part of providers to trust a biosimilar, incentives offered by the manufacturer of the original biologic, and lawsuits to prevent market entry. It is important to educate providers on the safety of biosimilars. A comprehensive strategy to facilitate the timely entry of cost-effective biosimilars can also help lower cost. In the United States, the FDA has approved 23 biosimilars. Success is mixed due to payer arrangements, but when optimized, these can be very successful. For example, in the case of filgrastim, there is over 60% adoption of the biosimilar, with a cost discount of approximately 30–40%^16^.

### Nonprofit generic companies

One way of lowering the cost of prescription drugs and to reduce drug shortages is nonprofit generic manufacturing. This can be set up and run by governments, or by nonprofit or philanthropic foundations. A recent example of such an endeavor is Civica Rx, a nonprofit generic company that has been set up in the United States.

### Compulsory licensing

Developed countries should be more willing to use compulsory licensing to lower the cost of specific prescription drugs when negotiations with drug manufacturers on reasonable pricing fail or encounter unacceptable delays. This process permitted under the Doha declaration of 2001, allows countries to override patent protection and issue a license to manufacture and distribute a given prescription drug at low cost in the interest of public health.

### Solutions: additional policy changes needed in the United States

The cost of prescription drugs in the United States is much higher than in other developed countries. The reasons for these are unique to the United States, and require specific policy changes.

### Value-based pricing

Unlike other developed countries, the United States does not negotiate over the price of a new drug based on the value it provides. This is a fundamental problem that allows drugs to be priced at high levels, regardless of the value that they provide. Thus, almost every new cancer drug introduced in the last 3 years has been priced at more than $100,000 per year, with a median price of approximately $150,000 in 2018. *The lack of value-based pricing in the United States also has a direct adverse effect on the ability of other countries to negotiate prices with manufacturers*. It greatly reduces leverage that individual countries have. Manufacturers can walk away from such negotiations, knowing fully well that they can price the drugs in the United States to compensate. A governmental or a nongovernmental agency, such as the Institute for Clinical and Economic Review (ICER), must be authorized in the United States by law, to set ceiling prices for new drugs based on incremental value, and monitor and approve future price increases. Until this is possible, the alternative solution is to cap prices of lifesaving drugs to an international reference price.

### Medicare negotiation

In addition to not having a system for value-based pricing, the United States has specific legislation that actually prohibits the biggest purchaser of oral prescription drugs (Medicare) from directly negotiating with manufacturers. One study found that if Medicare were to negotiate prices to those secured by the Veterans Administration (VA) hospital system, there would be savings of $14.4 billion on just the top 50 dispensed oral drugs^17^.

### Cap on price increases

The United States also has a peculiar problem that is not seen in other countries: marked price increases on existing drugs. For example, between 2012 and 2017, the United States spent $6.8 billion solely due to price increases on the existing brand name cancer drugs; in the same period, the rest of the world spent $1.7 billion *less* due to decreases in the prices of similar drugs^18^. But nothing illustrates this problem better than the price of insulin^19^. One vial of Humalog (insulin lispro), that costs $21 in 1999, is now priced at over $300. On January 1, 2020, drugmakers increased prices on over 250 drugs by approximately 5%^20^. The United States clearly needs state and/or federal legislation to prevent such unjustified price increases^21^.

### Remove incentive for more expensive therapy

Doctors in the United States receive a proportionally higher reimbursement for parenteral drugs, including intravenous chemotherapy, for more expensive drugs. This creates a financial incentive to choosing a more expensive drug when there is a choice for a cheaper alternative. We need to reform physician reimbursement to a model where the amount paid for drug administration is fixed, and not proportional to the cost of the drug.

### Other reforms

We need transparency on arrangements between middlemen, such as pharmacy-benefit managers (PBMs) and drug manufacturers, and ensure that rebates on drug prices secured by PBMS do not serve as profits, but are rather passed on to patients. Drug approvals should encourage true innovation, and approval of marginally effective drugs with statistically “significant” but clinically unimportant benefits should be discouraged. Importation of prescription drugs for personal use should be legalized. Finally, we need to end direct-to-patient advertising.

## Solutions that can be implemented by physicians and physician organizations

Most of the changes discussed above require changes to existing laws and regulations, and physicians and physician organizations should be advocating for these changes. It is disappointing that there is limited advocacy in this regard for changes that can truly have an impact. The close financial relationships of physician and patient organizations with pharmaceutical companies may be preventing us from effective advocacy. We also need to generate specific treatment guidelines that take cost into account. Current guidelines often present a list of acceptable treatment options for a given condition, without clear recommendation that guides patients and physicians to choose the most cost=effective option. Prices of common prescription drugs can vary markedly in the United States, and physicians can help patients by directing them to the pharmacy with the lowest prices using resources such as goodrx.com^22^. Physicians must become more educated on drug prices, and discuss affordability with patients^23^.

## References

[1] IQVIA. The global use of medicine in 2019 and outlook to 2023. https://www.iqvia.com/insights/the-iqvia-institute/reports/the-global-use-of-medicine-in-2019-and-outlook-to-2023 (Accessed December 27, 2019).

[2] IQVIA. Global oncology trends 2019. https://www.iqvia.com/insights/the-iqvia-institute/reports/global-oncology-trends-2019 (Accessed December 27, 2019).

[3] Kamal, R., Cox, C. & McDermott, D. What are the recent and forecasted trends in prescription drug spending? https://www.healthsystemtracker.org/chart-collection/recent-forecasted-trends-prescription-drug-spending/#item-percent-of-total-rx-spending-by-oop-private-insurance-and-medicare_nhe-projections-2018-27 (Accessed December 31, 2019).

[4] Siddiqui M, Rajkumar SV (2012). The high cost of cancer drugs and what we can do about it. Mayo Clinic Proc..

[5] Kantarjian H, Rajkumar SV (2015). Why are cancer drugs so expensive in the United States, and what are the solutions?. Mayo Clinic Proc..

[6] Rajkumar, S. V. The high cost of insulin in the united states: an urgent call to action. *Mayo Clin. Proc.*; this issue (2020). 10.1016/j.mayocp.2019.11.013 31902423

[7] DiMasi JA, Grabowski HG, Hansen RW (2016). Innovation in the pharmaceutical industry: new estimates of R&D costs. J. Health Econ..

[8] Almashat, S. Pharmaceutical research costs: the myth of the $2.6 billion pill. https://www.citizen.org/news/pharmaceutical-research-costs-the-myth-of-the-2-6-billion-pill/ (Accessed December 31, 2019) (2017).

[9] Prasad V, Mailankody S (2017). Research and development spending to bring a single cancer drug to market and revenues after approval. JAMA Intern. Med..

[10] Scutti, S. Big Pharma spends record millions on lobbying amid pressure to lower drug prices. https://www.cnn.com/2019/01/23/health/phrma-lobbying-costs-bn/index.html (Accessed December 31, 2019).

[11] Amin, T. Patent abuse is driving up drug prices. https://www.statnews.com/2018/12/07/patent-abuse-rising-drug-prices-lantus/ (Accessed November 16, 2019).

[12] Hancock, J. & Lupkin, S. Secretive ‘rebate trap’ keeps generic drugs for diabetes and other ills out of reach. https://khn.org/news/secretive-rebate-trap-keeps-generic-drugs-for-diabetes-and-other-ills-out-of-reach/ (Accessed November 16, 2019).

[13] Feldman, R. ‘One-and-done’ for new drugs could cut patent thickets and boost generic competition. https://www.statnews.com/2019/02/11/drug-patent-protection-one-done/ (Accessed December 31, 2019).

[14] Cohen, M. et al. Policy options for increasing generic drug competition through importation. Health Affairs Blog https://www.healthaffairs.org/do/10.1377/hblog20190103.333047/full/ (Accessed November 16, 2019).

[15] Bennett CL (2014). Regulatory and clinical considerations for biosimilar oncology drugs. Lancet Oncol.

[16] Fein, A. J. We shouldn’t give up on biosimilars—and here are the data to prove it. https://www.drugchannels.net/2019/09/we-shouldnt-give-up-on-biosimilarsand.html (Accessed December 31, 2019).

[17] Venker B, Stephenson KB, Gellad WF (2019). Assessment of spending in medicare part D if medication prices from the department of veterans affairs were used. JAMA Intern. Med..

[18] IQVIA. Global oncology trends 2018. https://www.iqvia.com/insights/the-iqvia-institute/reports/global-oncology-trends-2018 (Accessed January 2, 2018).

[19] Prasad, R. The human cost of insulin in America. https://www.bbc.com/news/world-us-canada-47491964 (Accessed November 16, 2019).

[20] Erman, M. More drugmakers hike U.S. prices as new year begins. https://www.reuters.com/article/us-usa-healthcare-drugpricing/more-drugmakers-hike-u-s-prices-as-new-year-begins-idUSKBN1Z01X9 (Accessed January 3, 2020).

[21] Anderson, G. F. It’s time to limit drug price increases. Health Affairs Blog. https://www.healthaffairs.org/do/10.1377/hblog20190715.557473/full/ (Accessed November 16, 2019).

[22] Gill, L. Shop around for lower drug prices. Consumer Reports 2018. https://www.consumerreports.org/drug-prices/shop-around-for-better-drug-prices/ (Accessed November 16, 2019).

[23] Warsame R (2019). Conversations about financial issues in routine oncology practices: a multicenter study. J. Oncol. Pract..

